# Effect of traffic-related air pollution on cough in adults with polymorphisms in several cough-related genes

**DOI:** 10.1186/s12931-022-02031-8

**Published:** 2022-05-04

**Authors:** Michael Yoon, Min Hyung Ryu, Ryan D. Huff, Maria G. Belvisi, Jaclyn Smith, Chris Carlsten

**Affiliations:** 1 Air Pollution Exposure Laboratory, Division of Respiratory Medicine, Department Medicine, Vancouver Coastal Health Research Institute, The University of British Columbia, Vancouver, BC Canada; 2 Research and Early Development, Respiratory Pharmacology Group, Respiratory & Immunology, BioPharmaceuticals R&D, AstraZeneca, Gothenburg, Sweden; 3 Airway Disease, National Heart and Lung Institute, Imperial College London, London, UK; 4 Division of Infection, Immunity, and Respiratory Medicine, University of Manchester, 16 Manchester Academic Health Sciences Centre, and Manchester University NHS Foundation 17 Trust, Manchester, UK; 5 The Lung Center, Vancouver General Hospital—Gordon and Leslie Diamond Health Care Centre, 2775 Laurel St. 7th Floor, Vancouver, BC V5Z 1M9 Canada

**Keywords:** Cough, Air pollution, Gene-by-environment interaction

## Abstract

With prevalent global air pollution, individuals with certain genetic predispositions and sensitivities are at of higher risk of developing respiratory symptoms including chronic cough. Studies to date have relied on patient-filled questionnaires in epidemiological studies to evaluate the gene-by-environment interactions. In a controlled human exposure study, we evaluated whether genetic risk score (GRS) based on cough-related single-nucleotide polymorphisms (SNPs) are associated with a cough count over 24 h post-exposure to diesel exhaust (DE), a model for traffic-related air pollution. DE is a mixture of several known air pollutants including PM_2.5_, CO, NO, NO_2_, and volatile organic compounds. Under closely observed circumstances, we determined that GRS constructed from 7 SNPs related to TRPA1, TRPV1, and NK-2R were correlated with cough count. Selection of channels were based on prior knowledge that SNPs in these channels lead to acute airway inflammation as a result of their increased sensitivity to particulate matter. We performed a linear regression analysis and found a significant, positive correlation between GRS and cough count following DE exposure (p = 0.002, R^2^ = 0.61) and filtered air (FA) exposure (p = 0.028, R^2^ = 0.37). Although that correlation was stronger for DE than for FA, we found no significant exposure-by-GRS interaction. In summary, cough-relevant GRS was associated with a higher 24 h cough count in a controlled setting, suggesting that individuals with a high GRS may be more susceptible to developing cough regardless of their exposure. The trend towards this susceptibility being more prominent in the context of traffic-related air pollution remains to be confirmed.

*Trial registration:* ClinicalTrial.gov NCT02236039; NCT0223603. Registered on August 11, 2014, https://clinicaltrials.gov/ct2/show/NCT02236039.

## Background

Cough is the body’s protective mechanism in response to irritants in the respiratory tract, but persistent cough in patients with respiratory conditions compromises their quality of life. Single nucleotide polymorphisms (SNPs) in several cough-related receptor genes are associated with respiratory conditions, including asthma and chronic cough [1–3].

Cough can be elicited by traffic-related air pollution (TRAP) activating airway nerves expressed along the respiratory tract [4]. Transient receptor potential vanilloid-1 (TRPV1) and transient receptor potential ankyrin-1 (TRPA1) are ionic channels that particulate matter can bind to and modulate airway inflammation and cough [4, 5]. A variant of the neurokinin-2 receptor (NK-2R) may modulate cough sensitivity because diesel exhaust (DE) particles can bind to *NK-2R* to induce inflammation by increasing plasma extravasation [6, 7].

Candidate gene analysis studies and in vitro experiments modulating TRP channels suggest SNPs in *TRPV1* and *TRPA1* are linked to childhood asthma and chronic cough [1, 2, 8, 9]. To investigate the relationship between cough-related SNPs and cough count in the context of air pollution exposure, we used 24 h cough monitoring in a human exposure study to DE.

## Methods

All participants provided informed consent. The main (ClinicalTrial.gov ID: NCT02236039) and sub-study (i.e., cough monitoring) were approved by the University of British Columbia clinical research ethics board (H14-00821). In this randomized, double-blinded crossover study, 13 research participants aged 42–80 were monitored over 24 h following a 2 h exposure to DE (diluted to PM_2.5_ at a nominal concentration of 300 µg/m^3^) and filtered air (FA). FA was the sham control and had PM_2.5_ concentration 4.5 $\pm$ 4.2 µg/m^3^ vs 282.2 $\pm$ 44.4 µg/m^3^ in DE [10]. Detailed exposure parameters, study key dates, and eligibility criteria were detailed [10]. VitaloJAK™ ambulatory cough monitor measured cough counts [11]. SNP genotyping was performed using TaqMan™ SNP Genotyping Assays (Thermo Fisher Scientific). Control DNA sequences (Cornell Institute for Medical Research, Catalogue#: HG00103, HG00581, HG00654) were used for *TRPA1*, *NK-2R*, and *TRPV1*, respectively. DNA was amplified with TaqMan™ Genotyping MasterMix (Thermo Fisher Scientific) according to the manufacturer’s instructions. Genetic risk score (GRS) was calculated as the sum of 7 SNP risk alleles. SNPs were first selected based on their associations with cough and asthma based on previous literature [1, 2, 8, 9]. From the initial selection of 20 SNPs, those SNPs with a minor allele frequency, obtained from the 1000 Genome Browser on NCBI, greater than 0.25 were selected (excluding rs77038916). The risk allele for each SNP was: A for rs1384001; T for rs77038916; T for rs8065080; T for rs959974; G for rs222747; A for rs224534, and T for rs2277675 where the sum of the number of risk alleles would yield an unweighted GRS value (0 to 14). However, heterozygous individuals for rs77038916 (*NK-2R*) were not scored as the association model tested in the literature was recessive [7]. Because of our repeated measure design with outcomes that is non-independent and correlated, linear mixed-effects (LME) models were used for statistical comparison. Cough counts were log-transformed. In our LME models, the exposure, interaction term exposure-by-GRS, sex and age were fixed effects, and participant ID was the random effect. A linear regression was used to observe the relationship between GRS and cough count. All statistical analyses and plots generation was done using R (v.4.1.2, R foundation for Statistical Computing).

## Results

**Fig. 1:**
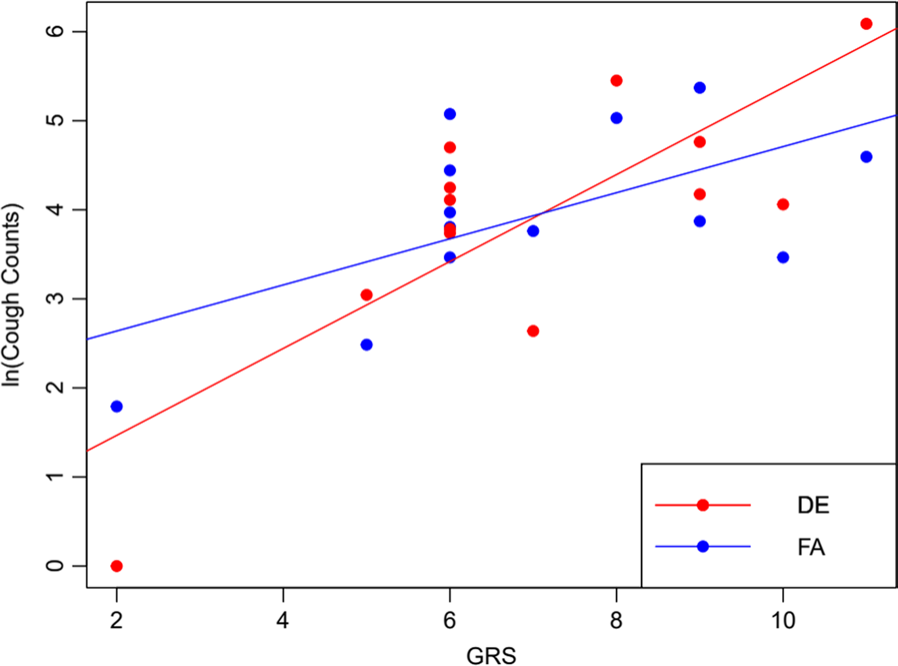
Relationship between genetic risk score (GRS) and cough counts for post-exposure to diesel exhaust (DE) or filtered air (FA). GRS was calculated as the unweighted sum of 7 single nucleotide polymorphism (SNP) risk alleles related to cough response. Risk alleles are described in methods

Participant characteristics and GRS for each participant are listed in Table 1. There was no significant effect of DE exposure on cough count, but linear regression analysis revealed an association between GRS and 24-h cough count (Fig. 1).

A significant and positive association was observed between GRS and cough count analyzing FA and DE data combined (p<0.001, R^2^=0.49). When exposures were analyzed separately, the association between GRS and cough count remained significant for DE (p=0.002, R^2^=0.61) and FA (p=0.028, R^2^=0.37). There was no significant exposure-by-GRS interaction on cough count despite this trend for GRS to be more positively associated with cough count upon exposure to DE than to FA.

## Discussion

Gene-environment interaction has been a focus in the field linking environmental exposure to chronic respiratory diseases such as asthma [12]. Understanding how underlying genetic variation influences cough in the context of environmental exposures can provide insight into how cough is regulated. Here we report a significant association between GRS constructed from SNPs related to *TRPV1*, *TRPA1* and *NK-2R* and 24-h cough count under closely observed circumstances. Our findings are novel in supporting established cough-associated SNPs in a clinical experimental setting and in suggesting that individuals with a higher number of risk alleles may have increased baseline sensitivity to PM_2.5_ based on a higher cough count.

Studies have linked multiple SNPs in *TRPV1* and *TRPA1* with asthma and cough. The Avon Longitudinal Study of Parents and Children found six SNPs significantly associated with childhood asthma development [9]. Furthermore, the Childhood Respiratory Health Study, the European Community Respiratory Health Survey, and the Epidemiological study on the Genetics and Environment of Asthma found *TRPV1* SNPs significantly associated with nocturnal, usual, and chronic cough [1, 8]. In support of the clinical data, experimental data have shown that these variants can modulate TRP channel activity resulting in altered calcium flux and inflammatory cytokine secretion causing a change in cough sensitivity [2, 8].

In this report, we note a pattern suggesting a greater influence on cough count by DE than FA given that the linear regression for DE had a stronger R^2^ coefficient with a more positive slope. To rule out that the association between GRS and cough count was driven by one SNP, we performed linear regressions between ln(cough count) and GRSs calculated with one SNP excluded in the GRS calculation. For cough count measured after DE exposure, there was an association between higher GRS and higher cough count, even when each SNP was removed. This association was weaker in the cough count measured after FA exposure, further supporting our hypothesis that GRS is more influential in the context of DE relative to FA. However, this hypothesis require independent validation study.

Sex as a potential effect modifier needs to be explored. We did not find significant exposure-by-sex (p = 0.63) or exposure-by-GRS-by-sex (p = 0.51) interaction. We recognize that a modest sample size limited our ability to test this interaction effect. There are also potential confounders including exposure to allergens and house dust. Another limitation is that participants were local to the Metro Vancouver area, limiting generalization to other regions with different genetic and environmental backgrounds. Furthermore, there are other SNPs in TRPV1, TRPA1, and NK-2R associated with cough or respiratory conditions not covered in our study (due to their low frequency in the general population).

## Conclusion

Unweighted GRS was associated with higher cough counts in a controlled setting, supporting previous observational literature connecting these mutations with cough, with a noteworthy trend in conferring a particular increase in cough when exposed to air pollution in the form of diesel exhaust.

**Table 1:** Participant characteristics

	Age (years)	Sex	GRS	Smoking history (pack-years)	Time since quitting smoking (years)	FEV_1_/FVC	FEV1 (L)	FEV1 (% Predicted)	DE cough count	FA cough count
1	42	F	12	0	–	86	2.41	92	440	98
2	56	F	6	0	–	81	2.61	103	43	31
3	58	M	8	0	–	75	4.46	133	232	152
4	72	F	9	0	–	80	3.31	162	64	47
5	50	M	6	15	16	79	4.05	106	41	44
6	56	M	5	50	8	74	3.43	113	20	11
7	70	M	6	90	19	72	2.52	93	69	52
8	66	F	7	24	16	54	1.87	86	13	42
9	67	M	2	52.5	11	49	2.62	76	0	5
10	70	F	6	12	33	48	1.79	88	109	159
11	70	M	9	19.5	16	64	2.44	89	116	214
12	75	M	6	7	41	67	3.11	116	60	84
13	80	M	10	10	30	54	2.33	97	57	31

Lung function data were measured after a bronchodilator (salbutamol) use during in-person screening. All participants were not current smokers. One pack-year is equivalent to smoking 1 pack (20 cigarettes) of cigarette every day for one year

*M* Male, *F* Female, *GRS* genetic risk score, *COPD* chronic obstructive pulmonary disease, *FEV*_*1*_*/FVC* ratio of forced expiratory volume in one second to functional vital capacity; FEV_1_% predicted, percent of predicted forced expiratory volume in 1 s; *N/A* not applicable

## Data Availability

The datasets used and/or analysed during the current study are available from the corresponding author on reasonable request.

## Abbreviations

SNP: Single nucleotide polymorphism
TRAP: Traffic-related air pollution
GRS: Genetic risk score
DE: Diesel exhaust
FA: Filtered air
TRPA1: Transient receptor potential ankyrin-1
TRPV1: Transient receptor potential vanilloid-1
NK-2R: Neurokinin-2 receptor
